# Human-Centered Design Strategies for Device Selection in mHealth Programs: Development of a Novel Framework and Case Study

**DOI:** 10.2196/16043

**Published:** 2020-05-07

**Authors:** Ashley Marie Polhemus, Jan Novák, Jose Ferrao, Sara Simblett, Marta Radaelli, Patrick Locatelli, Faith Matcham, Maximilian Kerz, Janice Weyer, Patrick Burke, Vincy Huang, Marissa Fallon Dockendorf, Gergely Temesi, Til Wykes, Giancarlo Comi, Inez Myin-Germeys, Amos Folarin, Richard Dobson, Nikolay V Manyakov, Vaibhav A Narayan, Matthew Hotopf

**Affiliations:** 1 Merck Research Labs Information Technology Merck Sharpe & Dohme Prague Czech Republic; 2 Epidemiology, Biostatistics and Prevention Institute University of Zürich Zürich Switzerland; 3 Department of Anthropology and Human Genetics Faculty of Science Charles University Prague Czech Republic; 4 Institute of Psychiatry, Psychology and Neuroscience King's College London London United Kingdom; 5 Neurology Services San Raffaele Hospital Multiple Sclerosis Centre Milan Italy; 6 Department of Engineering and Applied Science University of Bergamo Bergamo Italy; 7 National Institute for Health Research Maudsley Biomedical Research Centre South London and Maudsley NHS Foundation Trust London United Kingdom; 8 Patient Advisory Board Remote Assessment of Disease and Relapse Research Program King's College London London United Kingdom; 9 Merck Research Labs Information Technology Merck Sharpe & Dohme Singapore Singapore; 10 Pharmacokinetics, Pharmacodynamics, and Drug Metabolism Merck & Co, Inc Kenilworth, NJ United States; 11 Department for Neurosciences Center for Contextual Psychiatry Katholieke Universiteit Leuven Leuven Belgium; 12 Janssen Pharmaceutica NV Beerse Belgium; 13 Research and Development Information Technology Janssen Research & Development, LLC Titusville, NJ United States

**Keywords:** human-centric design, design thinking, patient centricity, device selection, technology selection, remote patient monitoring, remote measurement technologies

## Abstract

**Background:**

Despite the increasing use of remote measurement technologies (RMT) such as wearables or biosensors in health care programs, challenges associated with selecting and implementing these technologies persist. Many health care programs that use RMT rely on commercially available, “off-the-shelf” devices to collect patient data. However, validation of these devices is sparse, the technology landscape is constantly changing, relative benefits between device options are often unclear, and research on patient and health care provider preferences is often lacking.

**Objective:**

To address these common challenges, we propose a novel device selection framework extrapolated from human-centered design principles, which are commonly used in de novo digital health product design. We then present a case study in which we used the framework to identify, test, select, and implement off-the-shelf devices for the Remote Assessment of Disease and Relapse-Central Nervous System (RADAR-CNS) consortium, a research program using RMT to study central nervous system disease progression.

**Methods:**

The RADAR-CNS device selection framework describes a human-centered approach to device selection for mobile health programs. The framework guides study designers through stakeholder engagement, technology landscaping, rapid proof of concept testing, and creative problem solving to develop device selection criteria and a robust implementation strategy. It also describes a method for considering compromises when tensions between stakeholder needs occur.

**Results:**

The framework successfully guided device selection for the RADAR-CNS study on relapse in multiple sclerosis. In the initial stage, we engaged a multidisciplinary team of patients, health care professionals, researchers, and technologists to identify our primary device-related goals. We desired regular home-based measurements of gait, balance, fatigue, heart rate, and sleep over the course of the study. However, devices and measurement methods had to be user friendly, secure, and able to produce high quality data. In the second stage, we iteratively refined our strategy and selected devices based on technological and regulatory constraints, user feedback, and research goals. At several points, we used this method to devise compromises that addressed conflicting stakeholder needs. We then implemented a feedback mechanism into the study to gather lessons about devices to improve future versions of the RADAR-CNS program.

**Conclusions:**

The RADAR device selection framework provides a structured yet flexible approach to device selection for health care programs and can be used to systematically approach complex decisions that require teams to consider patient experiences alongside scientific priorities and logistical, technical, or regulatory constraints.

## Introduction

When used as part of health care programs, remote measurement technologies (RMT) such as wearables or biosensors have the potential to affect clinical decision making, provide novel health insights, and improve the standard of care in a variety of disease areas [1-4]. RMT is a subset of mobile health (mHealth) technologies, which includes “any technology that enables monitoring of a person’s health status through a remote interface, which can then be transmitted to a healthcare provider” for review or as a means of education for the user themselves [5]. Though use of RMT in health care programs has grown in recent years [1,2,6,7], its impact on health outcomes does not always live up to its supposed potential [1,7,8].

Successful utilization of RMT depends on careful consideration of the program’s scientific, technical, and usability requirements. However, many programs employ commercially available, “off-the-shelf” devices that cannot be customized according to these requirements. In such cases, program designers are challenged to select devices from hundreds of options [9] in a marketplace where validation is sparse [1,7,8], product turnover is high [10], and relative benefits between device options are often unclear. Comparative studies show either limited accuracy or low to moderate agreement between similar, widely-used devices for common measurements such as activity levels [11-14], sleep [14-16], heart rate [12,17,18], and energy expenditure [14,16,19]. Few industry-wide data standards have been established [6,9,20], and different devices may define and report measurements in ways that are not directly comparable [13]. Additionally, the experiences of potential users—including patients, caregivers, and health care professionals—affect the use of RMT heavily [21-23], but these insights are often not collected or transformed into technology requirements [24]. Unfortunately, RMT that do not cater to user needs can increase patient, caregiver, and health care provider burden in otherwise promising health care programs [6,25] and may negatively impact enrollment and retention [26].

Those designing health care programs often struggle to navigate device selection due to the technology landscape’s complexity and potential tensions between device selection criteria [4,20,27]. To date, few best practices exist to guide the selection of off-the-shelf devices. The Framework of Specifications to Consider During Mobile Technology Selection developed by the Clinical Trial Transformation Initiative lists factors to consider when selecting RMT, including technical performance, data management, safety, and human factors [28]. However, it does not provide a method to apply or prioritize these factors. The Digital Health Selection Framework by the Institute for Healthcare Improvement [29] describes a computational method for assessing the technology landscape based on high-level selection criteria. However, this framework aims to support the development of health care policy, and the method does not support the identification and ranking of sufficiently detailed requirements for use in individual program designs. Scientific publications provide only high-level commentary on device selection, suggesting that designers consider technical requirements, user experiences, data quality, safety, privacy, regulations, costs, and other factors when choosing technologies [27-30]. Such publications also discuss the need to set detailed objectives [27,31] and gather requirements from a diverse set of stakeholders [24,28,31]. However, to our knowledge, no publication describes systematic methods for gathering, prioritizing, and weighing device selection criteria within the context of the program’s users, environments, and goals.

This is problematic, as device-related factors have the potential to limit the success, reproducibility, or scalability of otherwise promising health care programs. In this study we propose a framework to guide device selection based on human-centered design (HCD) principles. We then demonstrate the use of this framework in a research program using RMT to identify and predict relapses in multiple sclerosis (MS). 

## Methods

### Human-Centered Design in Mobile Health

HCD is increasingly used to design novel health care programs and products [4,10,32-37]. HCD is a series of methods through which designers study a product user’s needs and environment and then design accordingly [38,39]. Designers engage or “empathize” with potential users then generate ideas, develop prototypes, and test those prototypes with the people for whom they are designing [38,39]. Designers alternate between divergent and convergent thinking, looking broadly to understand context and possible solutions, and then converging onto a final problem statement, approach, or solution [38,40]. Many methods also employ agile or lean principles, which use rapid prototyping, feedback loops, and learning cycles to drive an iterative design and implementation process [38,41]. These methods allow designers to develop a deep understanding of the contextual factors that affect design, making them well-suited to support product design in complex, ambiguous, and rapidly-changing environments. The merits of HCD in health care program design have been discussed at length elsewhere [24,33], though such methods are largely applied to de novo designs, rather than technology selection.

HCD frameworks exist for a variety of mHealth applications, including behavioral intervention design [32], implementation of patient-facing technology in interventional clinical trials [31], mHealth solution development and validation [10,33,42], stakeholder engagement [36], and requirement development [43]. Though these frameworks are inconsistent in their language, they employ a set of common methods to inform the design of digital solutions within the context of the health care system (Textbox 1).

**Common human-centered design principles recommended in mobile health solution design.**

• Assemble a multidisciplinary team [31,43]

• Iterate throughout the design process [10,31-34,36,42,43]

• Begin by conducting stakeholder engagement activities to understand users’ needs and environments [31-34,36,42,43]

• Conduct ideation sessions in which a variety of approaches and potential solutions are explored [10,31,32,34,42]

• Enable a variety of stakeholders, including patients, health care professionals, technical experts, and others to participate in the design process [31-34,36,42]

• Prioritize identified requirements and resolve conflicting requirements through further engagement with team members and stakeholders [43]

• Prototype and test with end users prior to scaled implementation [10,31-34,42,43]

• Consider the implementation strategy early and refine it during the design process [31-33]

• Measure the solution’s impact and efficacy [10,31,43]

• Share both positive and negative lessons learned with relevant stakeholders to improve current and future designs [31,32]

To our knowledge, no HCD framework addresses the challenges associated with selecting off-the-shelf devices for digital health care interventions. We hypothesized that HCD methods may also be useful for that purpose, because HCD methods address similar design challenges to those posed by device selection. Such challenges include understanding and navigating complicated contextual factors [31,32,34,42,43], engaging with multifunctional stakeholders [36], and prioritizing requirements while addressing diverse stakeholder needs [43].

### RADAR Device Selection Framework

**Figure 1:**
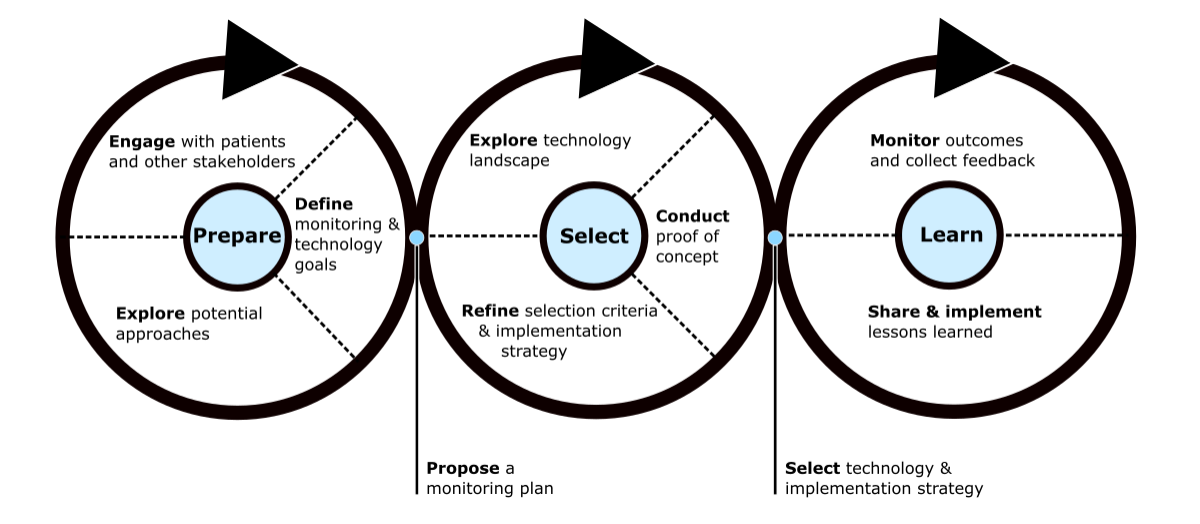
RADAR device selection framework.

A novel device selection method was developed for the Remote Assessment of Disease and Relapse-Central Nervous System (RADAR-CNS) project, a collaborative research program using RMT to study central nervous system disease progression. This framework was developed empirically based on the authors’ previous experience with HCD in medical technology design. We hypothesized that HCD methods could help design teams manage the complexity inherent to device selection. Therefore, the three-stage RADAR-CNS device selection framework (Figure 1) was proposed and optimized for the RADAR-CNS program. The framework uses HCD techniques to explore the technology landscape, refine device requirements, develop an implementation strategy, and make informed decisions in parallel with program design and implementation.

### Stage 1: Prepare

In this stage, the team studies contextual and user-related factors that may affect device use and implementation. The goals of the program, motivations and experiences of patients, involvement of caregivers, and symptoms or sensitivities related to the target disease area will define how user-friendly, discreet, configurable, or multifunctional a device must be. These activities are analogous to the empathize, define, and ideate steps of the design thinking process [44], and similar steps have been proposed in other frameworks [32,33]. In stage 1 we highlight relevant device-related insights that can be collected through HCD methods early in the program planning process.

#### Engage With Patients and Other Stakeholders

Simblett et al (2018) [22] described five categories of facilitators and barriers that influence patient engagement with RMT: health status, usability, convenience and accessibility, perceived utility, and motivation. During the preparation stage, the device selection team engages with patients and other stakeholders to explore these factors, identify user needs, and draft technology requirements. These activities can be conducted alongside other engagement activities designed to inform program goals or design. Methods for engaging with these and other relevant stakeholders have been proposed, including co-design sessions, focus groups, interviews, workshops, and surveys [44-48]. Integrated patient advisory boards can also guide discussions and decisions throughout the device selection process.

Though published literature on research priorities and usability requirements may provide general insights into patient perspectives in a variety of disease areas [22,24,49], primary research with the program’s target population is critical [4,24]. RMT can increase the burden associated with giving and receiving care [4,9,25], which must be minimized to enable sustained program adoption. Direct engagement with potential users provides the nuanced insights that are necessary to minimize burden and increase the chances of program success. Patients may be the primary users of the technology; however, caregivers, health care professionals, and others should also be engaged, as they affect patients’ willingness and motivation to engage with RMT [22].

#### Explore Potential Approaches

The team then explores different approaches for measuring health status. Options should reflect scientific and clinical goals as well as patients’ priorities. The team should propose potential *measurement schemes* that list relevant variables or outcomes, surrogate measurements, data streams, required sensors, and desired frequency of measurements. In this stage, it is helpful to use good brainstorming techniques such as those described in IDEO’s Design Thinking Bootleg [44] to generate a variety of options and encourage creativity by limiting discussion of potential constraints. The team should define potential program goals, endpoints, and measurement schemes before exploring technology options and implementation strategies [20,27,31]. Delaying discussion of specific technology options forces the team to frame device selection around program and user needs, thereby preventing the design of a program around a familiar but ill-suited technology.

#### Define Measurement and Technology Goals

Based on the outcomes of the engagement and brainstorming activities, the team should converge on one or more promising measurement schemes and clearly define goals for the RMT. Only once these are defined should the team draft selection criteria. The team should clearly state what compromises they are and are not willing to make, as these choices will drive final device selection. Examples of relevant device selection criteria have been published elsewhere [27-29].

#### Milestone 1: Propose a Monitoring Plan

By the end of this stage, the team should have developed a robust understanding of stakeholder needs and priorities, a well-defined program goal, one or more potential measurement schemes, and a preliminary understanding of the technology landscape and technology selection criteria. The activities that led to this preliminary plan will provide necessary context to support device selection decisions, especially when no device meets all criteria and concessions must be made. To achieve this level of clarity, the team may need to conduct multiple iterations of the “Prepare” stage. For example, the team may need to re-engage stakeholders to confirm the acceptability of a measurement scheme and then adjust the scheme in subsequent brainstorming activities.

### Stage 2: Select

In this stage, the team progresses iteratively through a series of activities to identify a suitable device and refine an implementation strategy. With each iteration, the team should identify and answer outstanding questions, refine their thinking, and add detail to their proposed implementation plan. The team should first think broadly before refining the measurement scheme and implementation plan to reflect the program’s constraints. This approach allows the team to explore multiple approaches efficiently and to pursue creative options for getting as close to an *ideal* solution as possible.

#### Explore Technology Landscape

First, the team performs an initial technology landscape assessment and compiles a list of potentially suitable technologies. Devices should then be systematically excluded from this list based on user feedback and updates to the selection criteria or measurement scheme. When appropriate, additional options should be added to reflect updates to the selection criteria and implementation strategy. A *short list* of candidates should be defined based on the team’s selection criteria.

#### Refine Selection Criteria and Implementation Strategy

Based on identified technology options and insights from user engagement, the team should begin to define how the technology will be implemented. Factors such as the necessary connections to information technology (IT) systems, device provisioning, training, frequency of device use, compliance monitoring, and data syncing methods should be considered. This strategy may change over time; however, considering these factors early in the selection process will help the team understand potential infrastructure or logistical constraints that could impact device selection. Lack of such strategic planning has been shown to hinder successful implementation of RMT [30].

Off-the-shelf devices may not fit the initial measurement scheme and selection criteria perfectly. Iterative refinement of the selection criteria, measurement scheme, implementation strategy, and technology landscape will help the team explore creative alternatives, make minor concessions, and identify a small group of candidate technologies that meet most criteria.

#### Conduct Proof of Concept

Throughout this process, additional questions about candidate devices’ characteristics and relative advantages are likely to emerge. In the proof of concept (PoC) phase, the team should conduct targeted tests to answer these questions. PoCs are targeted device assessments that can be conducted quickly prior to implementation in a clinical study that enable rapid learning and decision making during the technology selection process [4,31]. PoCs can test technical characteristics (eg, bench testing for data quality, connectivity, durability), assess user experience in the target population (eg, usability studies), compare candidate devices, or test aspects of a technology’s implementation strategy (eg, “dry runs” to test training protocols and technology support systems) [31]. The results of any PoC should be actionable, either in a technology selection decision or to influence refinement of the implementation strategy.

#### Milestone 2: Select Technology and Implementation Strategy

By the end of this stage, the team should have narrowed the landscape to a few well-defined technology options, though each is likely to require compromise. To weigh these options, the team should use a systematic method to compare candidate devices and their required compromises. The team should facilitate multifunctional conversations to develop understanding of the required compromises and consensus on a final decision. The team should also finalize an implementation strategy, validating it through PoC testing and additional user feedback as necessary.

### Stage 3: Learn

#### Monitor Outcomes and Collect Feedback

The team should also devise mechanisms to collect feedback, experiential data, opportunities for improvement, and opportunities for learning from active programs, and these mechanisms should be included in research protocols if appropriate. Validated questionnaires such as the Post-Study System Usability Questionnaire [50] or the Technology Assessment Model [51] are widely used, and additional quantitative metrics such as device use or help desk engagement rates may also provide insights. Qualitative interviews with patients and health care professionals can identify specific opportunities to improve the implementation strategies, training materials and methods, technologies, or technology support systems.

#### Share and Implement Lessons Learned

The design and learning processes should not stop when the program is launched [30]. Quantitative, qualitative, and experiential data collected during all three stages of the framework should be used to continually refine the implementation strategy to ensure efficacy, efficiency, user engagement, ease of use, and clinical utility. In the case of a clinical study where continuous adjustments to the implementation strategy may jeopardize a program’s scientific goals, feasibility studies or clinical process evaluations may be used to test and refine the implementation strategy [4,20,52]. Sometimes, devices or technologies selected for an investigational system may not be practical for use in a scaled clinical practice. In this case, appropriate technologies should be selected or designed to fit the system requirements that were collected during investigational implementation. Both positive and negative findings should be shared to inform technology selection decisions in future programs.

## Results

### RADAR-CNS Case Study

RADAR-CNS is a public-private research program leveraging RMT to develop new ways of assessing disease progression in depression, epilepsy, and MS [53]. The RADAR Device Selection framework was used to select devices for several RADAR-CNS studies; however, only its use in a study on MS disease progression is explored here. In this 2-year study, wearable devices and a custom application collect longitudinal health-related data from people with relapsing-remitting MS. The aim is to develop algorithms that can predict relapse and improve patient care. Details of the study’s full protocol are outside the scope of this publication, and only device selection procedures are described here.

### RADAR-CNS: Prepare

A cross-functional team of clinicians, researchers, and technical experts was established, and RADAR-CNS’ patient advisory board [54] was also regularly consulted. We worked with people living with MS to understand their perspectives on research priorities, usability requirements, desired device features, and factors influencing sustained engagement with RMT. We conducted a systematic literature review to identify relevant discussion topics [22] and initiated a series of surveys and semistructured focus groups for people living with MS to identify factors affecting engagement with RMT [55]. Participants provided feedback on preferred device features and engagement schemes as well as perspectives on value and privacy. Much of this work has been published previously [55-57]. Participants emphasized the importance in accommodating MS symptoms, making the system easily usable, and enabling users to exert control within the RMT system [55].

We then explored areas of scientific research priority, including cognition, mood, physical activity, sleep, social interactions, speech, and stress. We identified variables that aligned with patient and scientific research priorities, discussed potential measurement schemes, and began to research technological options (eg, data streams, sensors, active tasks, analytical methods). We also began to discuss a variety of technical, user experience, regulatory, and other considerations relevant to the research program. These are described in Multimedia Appendix 1.

#### Milestone 1: Propose a Monitoring Plan

We prioritized the identified variables based on clinical utility, technological feasibility, alignment with patient priorities, and ethical considerations to select a final measurement scheme for the biosensors (Table 1). Additional clinical, traditional, and mobile data collection methods were also selected, but are outside the scope of this case study. Based on this scheme and patient insights, we defined a preliminary list of required and desired device selection criteria, their relative priorities, and opportunities for compromise. Briefly, the criteria described desired technical capabilities, data quality, user experience, regulatory status, privacy, required investment, and vendor characteristics. Opportunities for compromise included conditions under which multiple devices could be used, acceptable concessions described by patients, and acceptable trade-offs to meet the study budget (eg, willingness to develop bespoke software if device costs are reduced). A summary of these criteria and compromises is available in Multimedia Appendix 1.

**Table 1:** Device-based remote measurement scheme for the RADAR-CNS multiple sclerosis study.

Factor	Measurement	Measurement Frequency
Gait	Measured via accelerometer and gyroscope during a 2-Minute Walk Test, tandem walk test, and normal daily activities	Clinical tests^a^, home tests^b^, free living^c^
Balance	Measured via accelerometer placed on the chest during Romberg’s Test and normal daily activities	Clinical tests, home tests, free living
Fatigue	Measured via heart rate variability and accelerometer during a 2-Minute Walk Test and normal daily activities	Clinical tests, home tests, free living
Heart rate and heart rate variability	Measured via one-lead electrocardiogram placed on chest during tests and normal daily activities	Clinical tests, home tests, free living
Heart rate and heart rate variability	Measured via photoplethysmography	Daily^d^
Sleep	Total sleep time and sleep patterns monitored via actigraphy or other mechanism	Daily
Daily Activity	Measured via actigraphy	Daily

^a^Clinical tests: once every 3 months.

^b^Home tests: once every 3 months.

^c^Free living: one week every 3 months.

^d^Daily: daily over the course of the study.

### RADAR-CNS: Select

We then identified relevant commercially-available consumer and research-grade devices. As no published database contained up-to-date information on available RMT, we conducted an online search and a literature search to identify devices that contained some or all of the sensors in the desired measurement scheme. This search yielded over 100 devices of various embodiments. Devices were systematically excluded through an iterative review process with clinical, analytical, and technical experts, during which potential technologies, priorities, and protocol adjustments were discussed. No single technology fulfilled all selection criteria; however, several devices that fulfilled *most* criteria were selected for further consideration either as stand-alone devices or for use in conjunction with other devices. These included the Fitbit Charge 2 (Fitbit, Inc., San Francisco, CA), the Withings Steel HR (Withings, Issy-les-Moulineaux, France), the Actigraph Link (ActiGraph LLC, Pensacola, FL), the Suunto Movesense sensor (Suunto Oy, Vantaa, Finland), the eMotion Faros 180 (Biomation, Ottawa, ON, Canada), and the MetaMotion R (MBIENTLAB Inc, San Francisco, CA).

#### Proof of Concept Testing

Questions regarding usability, data quality, and technical characteristics of the devices arose, prompting appropriate PoC testing of usability, technical features, and training procedures. This section describes two examples of these PoC tests and their impacts on technology selection.

##### Example: User Experience Proof of Concept

Sustained patient engagement with the devices was critical to the study’s success, because participants could be enrolled for up to 2 years. The patient advisory board participated in a workshop to provide feedback on candidate devices. Board members, including two members living with MS (authors JW and PB), interacted with each device and provided feedback on user-friendliness, technology preferences, potential impacts of MS symptoms on use, and suggestions for the implementation strategy. This feedback provided us with important context for prioritizing desired device characteristics. The board preferred adhesive patches over chest straps to affix chest-based devices and wrist-based wearables with a subtle or mainstream appearance. They also noted that any goals or feedback shown by the devices, such as daily activity counts, should be customizable. They voiced concern that displaying unrealistic goals could negatively impact participants’ motivation to engage with RMT or participate in the study, as people living with MS will almost certainly observe a decline in function over time.

##### Example: Technical Proof of Concept

Following a brainstorming session, the team decided to explore the option of sourcing sensors from an original equipment manufacturer. These devices would be less expensive and more customizable but required additional validation and configuration compared to other options. For commercial reasons, the identities of these devices are not shared. Data were collected from two devices to understand data structure, battery life, reliability of the Bluetooth connection, potential for data loss, data transfer requirements (eg, time, file size, memory availability), and device durability. The devices’ published specifications met the requirements; however, the testing demonstrated that neither device met study requirements. The first device’s data files were too large to sync more than a few hours of data over a Bluetooth connection, but the study required devices to sync data over Bluetooth outside the clinic. The second device did not meet battery life or data quality requirements in the desired configuration. We tested other candidate devices similarly to address the risks identified by the advisory board and the study teams.

In response to this PoC, we adjusted our technology landscape to include more expensive devices since the tested devices were the only two to meet original budget requirements. To accommodate this change, we also adjusted the implementation strategy to include logistics associated with device returns and reprovisions, thereby reducing the number of required devices and reducing the device cost per patient. This PoC did not yield positive results, but it allowed the team to make data-informed decisions on device candidates without compromising timelines or posing risks to the study.

#### Milestone 2: Select Technology and Implementation Strategy

Ultimately, we selected 2 devices to conduct all desired measurements. The eMotion Faros 180 was selected to monitor cardiac activity, gait, and balance during home-based active tasks and normal daily activities. The Fitbit Charge 2 was selected to monitor daily activity and sleep based on its superior user experience and battery life, as well as the precedence of Fitbit devices in MS programs [58-60], despite its inability to provide raw accelerometer data. Since no device containing an electrocardiogram, accelerometer, and gyroscope met the necessary criteria, data from the gyroscope sensor in participants’ cell phones were collected to identify turns during the 2-Minute Walk Test. A discussion guide used by the team to facilitate the final selection of the wrist-based device is included in Multimedia Appendix 2.

### RADAR-CNS: Learn

The RADAR-CNS study is ongoing at the time of publication. Surveys and interviews with participants are being conducted periodically throughout the study and device use rates will be monitored as the study progresses. Feedback will also be collected from investigators who conducted the studies. Insights gained through these interactions will be published at the end of the study and will be used to identify improvements to the measurement scheme, device selection, and implementation strategy before the system is available for use in clinical practice.

## Discussion

The RADAR-CNS Device Selection Framework provides methods to assess, prioritize, and adapt device selection criteria for health care programs according to stakeholder needs. The framework is presented linearly, but it is intended to be flexible so teams can move forward, backward, or repeat steps as needed to support device selection. In the RADAR-CNS study, we conducted several iterations of the Prepare and Select stages as our thinking evolved during the study design. These iterations enabled dialogue between the technical and clinical experts on the project, allowing us to establish common ground between stakeholders and ensure consensus on the final decision. We found that our success depended on the engagement of a multifunctional team during each stage of the framework, including investigators, IT specialists, data analysts, patients, health care professionals, and others. Each brought unique perspectives and needs to the process, and each ultimately made compromises to agree on a single technology and implementation strategy. To ensure alignment and mutual understanding between these stakeholders, it was important that members of the device selection team were skilled in “translating” clinical and technical requirements and their contexts for team members of diverse backgrounds.

Navigating complex stakeholder needs is one of the strengths of HCD, especially when program success is dependent on the willingness of people to continually engage with a technology. As its name suggests, HCD starts by asking designers to understand the people who will be using the technology [38,40,44]. It then enables designers to simultaneously explore program contexts and constraints, identifying connections and priorities between human and nonhuman factors [38,44]. In a systematic review of systematic reviews, Ross et al (2016) [30] found that early engagement with relevant stakeholders such as patients, clinicians, and others was important for successful mHealth implementation, and most frameworks for digital health care solution design echo that sentiment [33]. However, Altman et al (2018) [24] found that user engagement activities were frequently not conducted in such programs. Limited stakeholder centricity during program design and technology selection may ultimately threaten the program’s success. Poor user experiences caused by increased burdens [4,26], technical issues [22], lack of accommodations for health status [22], impersonal experiences [26], slowness [22,26], and poor or unclear interface designs [22] may cause patients to stop using the technology, or worse, drop out of the program. Altman et al [24] suggested that, by addressing user needs, HCD methods such as design thinking could increase uptake, adherence, and impact of health care programs that use RMT.

Here, we use HCD methods not to create new designs, but to identify which existing designs are best suited to a particular program. In the RADAR-CNS program, we used HCD methods to identify and prioritize a vast number of often conflicting needs and constraints, not only from patients but also from other “users” of the program: the clinicians caring for patients, the researchers studying diseases, and the technologists developing new monitoring tools. Many common HCD strategies such as empathizing with users, brainstorming, and iterative designing are present in this framework, making it compatible with other HCD approaches to program design or validation.

Though the RADAR Device Selection framework was implemented successfully in an observational research program, its validity in other settings, such as clinical trials of investigational therapies or interventional mHealth program design, must be established in future work. Examples of successful implementation of human-centered methods in health care and academic environments exist; however, their use is not yet routine. Such methods require a mindset shift, new skills, and adoption of additional study planning activities, with more time spent initially on stakeholder engagement [24].

Though selecting off-the-shelf devices for health care programs is often difficult, few best practices exist to guide program designers. To address this gap, we developed and successfully implemented the RADAR device selection framework, which incorporates HCD strategies into a three-stage approach for systematically identifying selection criteria, testing and selecting devices, and monitoring device-related outcomes. To improve RMT implementation in future programs, the methods used and lessons learned during device selection should be more routinely shared.

## Appendix

Multimedia Appendix 1 RADAR-CNS device selection considerations and selection criteria.

Multimedia Appendix 2 RADAR-CNS Multiple Sclerosis Study Device Selection Discussion Guide.

## Abbreviations

CNS: Central Nervous System
HCD: human-centered design
IT: information technology
mHealth: mobile health
MS: multiple sclerosis
PoC: proof of concept
RADAR: Remote Assessment of Disease and Relapse
RMT: remote measurement technologies.
