# Supplementary material for: Human-Centered Design Strategies for Device Selection in mHealth Programs: Development of a Novel Framework and Case Study
Source: JMIR Mhealth Uhealth. 2020 May 7;8(5):e16043. doi: 10.2196/16043 (PMC7243134; doi:10.2196/16043)
Supplement: Multimedia Appendix 1 [file mhealth_v8i5e16043_app1.docx]

# Multimedia Appendix 1: RADAR-CNS device selection considerations and selection criteria

Device selection considerations discussed throughout the development and evolution of selection criteria for the RADAR-CNS study fell into several major categories: technical specifications (Table S2.1), user experience (Table S2.2), data (Table S2.3), regulations and privacy, investment, and vendor considerations (Table S2.4). These considerations were used to develop selection criteria for the Multiple Sclerosis study (Table S2.5), including required criteria, desired criteria, and opportunities for compromise.

| **Table S2.1: Technical Considerations** | | |
| --- | --- | --- |
| **Hardware Features** | **Connectivity** | **Configurability** |
| Charge time | Method of data syncing (e.g., USB sync with cloud, direct sync with device) | Ability to customize technology/interface |
| Display on device | Syncing frequency | Ability to control what is displayed to patient |
| Battery life | OS/Mobile Device Compatibility | Sampling rate/data range |
| Ease of charging | Robustness of connection w/ mobile device | Ability to freeze firmware or software updates over the course of the study |
| Device form/placement | Data volume | SDK availability |
| Durability | API availability |  |

| **Table S2.2: User Experience Considerations** | | |
| --- | --- | --- |
| **Social Impact** | **Wear Limitations** | **Feedback & Motivation** |
| Users' self-perception when wearing the technology | Device comfort, feel of materials | Ability to present user with feedback |
| Social reaction to the technology | Device size | Does the device/feedback bring value to patients? |
| Therapeutic area sensitivity/stigma | Ability to put the device on, take the device off easily | Can the patients customize their goals and visible feedback? |
| Appearance - Does the device look cheap, expensive, medical, sporty, unprofessional, or ugly? | How does health status impact ability to use the technology? |  |
|  | Ability to wear during activities of daily living: work, sport, sleep, etc. |  |

| **Table S2.3: Data Considerations** | | |
| --- | --- | --- |
| **Quality** | **Access & Transparency** | **Standards & Format** |
| Measurement range | Accessibility of raw data | Is there a data standard? |
| Resolution, Precision, Accuracy | Open source vs. proprietary algorithms | Does the device adhere to data standards? |
| Sampling rate | Availability of annotations, contextual data, or metadata |  |
| Timestamp drift |  |  |
| Signal to noise ratio |  |  |
| Frequency & causes of data loss |  |  |

| **Table S2.4: Other Considerations** | | |
| --- | --- | --- |
| **Regulations & Privacy** | **Investment Required** | **Tech/Vendor Reliability** |
| Ability to import and use technology in appropriate geographical regions/locations | Translations of apps, interfaces, and instructions | Device maturity |
| Data privacy regulations: GDPR, HIPAA compliance | Required software & accessories | Company maturity/stability |
| Data storage location | Level of required development/customization | Vendor ability to scale (study size and geographically) |
| Is it, or does it need to be, an approved medical device? | Cost of devices, software, and other licensing | Vendor ability to provide tech support |
| Algorithm output: is the source and definition or each data point/stream clear? Does it need to be? | Availability of support & training materials | Validation/precedence in patient population |
| Can the data be encrypted? | Data management system development | Past use in clinical research |

| **Table S2.5: Summary of RADAR-CNS device selection criteria, priorities, and opportunities for compromise** | | | |
| --- | --- | --- | --- |
| **Category** | **Required Characteristics** | **Desired Characteristics** | **Priorities/Opportunities for Compromise** |
| Technical | - Minimum 24-hour battery life - Compatibility with Android OS - Reliable Bluetooth connectivity - API available for the app or cloud - Ability to upload data automatically outside the clinic at least weekly - Ability to store data when out of range of a Bluetooth connection   Chest-worn device   - Contains 3D accelerometer & 1-lead Electrocardiogram (ECG)   Wrist-worn device:   - Contains 3D accelerometer | - 5+ days battery life - Daily data upload - SDK available - Ability to limit access to features and data   Chest-worn device:   - Option for adhesive wear - Contains gyroscope   Wrist-worn device:   - Heart rate monitoring | Multiple devices may be used if no device contains all desired sensors |
| Data | Chest-worn device:   - Accelerometer: minimum 50 Hz sampling rate - ECG: minimum 250 Hz sampling rate - Minimal demonstrated data loss, noise, and movement artifact   Wrist-worn device:   - Maximum epoch length 15 minutes for activity levels, heart rate, and sleep | Wrist-worn device:   - Raw data available - Publicly available algorithm - Data structure & algorithm output comparable to other devices on the market - Ability to control/limit algorithm updates | Factors improving long-term patient engagement or compliance are of higher priority than raw data on the wrist-worn device |
| User Experience | - Participants should be able to don and operate devices despite symptoms of relapse, including dexterity loss, feeling loss in fingertips, and blurred vision - Devices should be discreet, and should not appear too cheap or too expensive - Devices should be comfortable and feasible to wear during most activities of daily living - Feedback should be provided to patients, who should be able to set their own goals - Device interfaces should be available in local languages | - Comfortable to wear during sleep - Accompanying apps are engaging to patients - Waterproof | Patients are willing to tolerate poorer user experience if the perceived quality of the data is higher  If devices cannot be worn comfortably during sleep, sleep monitoring will be excluded |
| Regulations, Safety, & Privacy | - Devices & infrastructure must meet all relevant local, national, and international privacy regulations - Devices should be safe for daily use | - CE-Mark for medical device classification | Wrist-worn consumer devices will be considered if they have precedence in an MS population |
| Investment | - Average cost of €150 euro per participant, inclusive of all devices | - Ability to acquire/deploy devices within 3 months | Additional IT development is acceptable to decrease cost.  Re-using devices is acceptable if only high-cost devices meet data quality requirements |
| Vendor | - High probability that devices/operating systems will be supported throughout the course of the 2-year study - Vendor viability and stability | - Ability to provide help desk support throughout study duration | Study sites can provide help desk support if required |
