# Supplementary material for: Human-Centered Design Strategies for Device Selection in mHealth Programs: Development of a Novel Framework and Case Study
Source: JMIR Mhealth Uhealth. 2020 May 7;8(5):e16043. doi: 10.2196/16043 (PMC7243134; doi:10.2196/16043)
Supplement: Multimedia Appendix 2 [file mhealth_v8i5e16043_app2.pptx]

## Slide 1
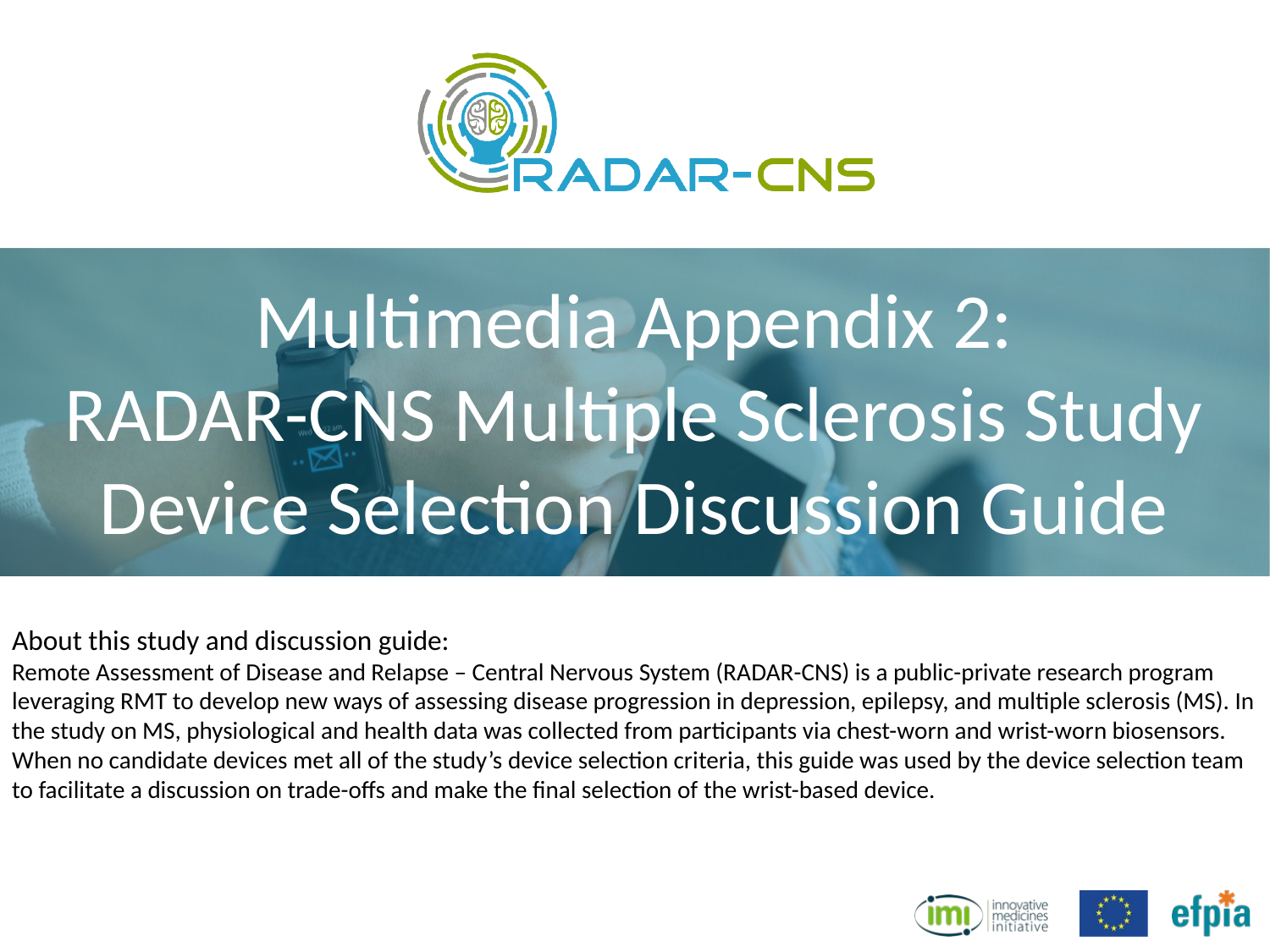

# Multimedia Appendix 2: RADAR-CNS Multiple Sclerosis Study Device Selection Discussion Guide
About this study and discussion guide:
Remote Assessment of Disease and Relapse – Central Nervous System (RADAR-CNS) is a public-private research program leveraging RMT to develop new ways of assessing disease progression in depression, epilepsy, and multiple sclerosis (MS). In the study on MS, physiological and health data was collected from participants via chest-worn and wrist-worn biosensors. When no candidate devices met all of the study’s device selection criteria, this guide was used by the device selection team to facilitate a discussion on trade-offs and make the final selection of the wrist-based device.
fgfd

## Slide 2
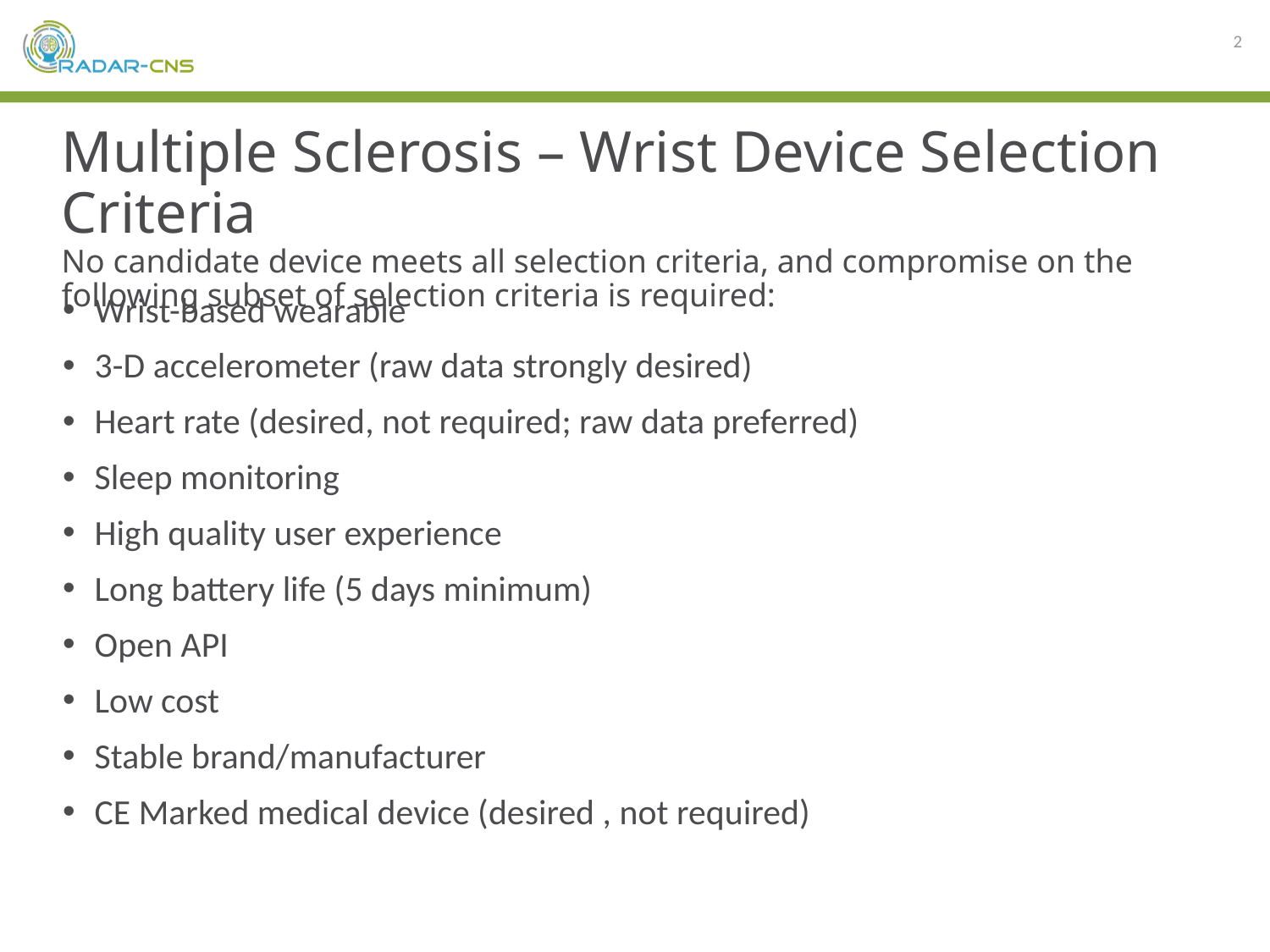

2
# Multiple Sclerosis – Wrist Device Selection Criteria No candidate device meets all selection criteria, and compromise on the following subset of selection criteria is required:
Wrist-based wearable
3-D accelerometer (raw data strongly desired)
Heart rate (desired, not required; raw data preferred)
Sleep monitoring
High quality user experience
Long battery life (5 days minimum)
Open API
Low cost
Stable brand/manufacturer
CE Marked medical device (desired , not required)

## Slide 3
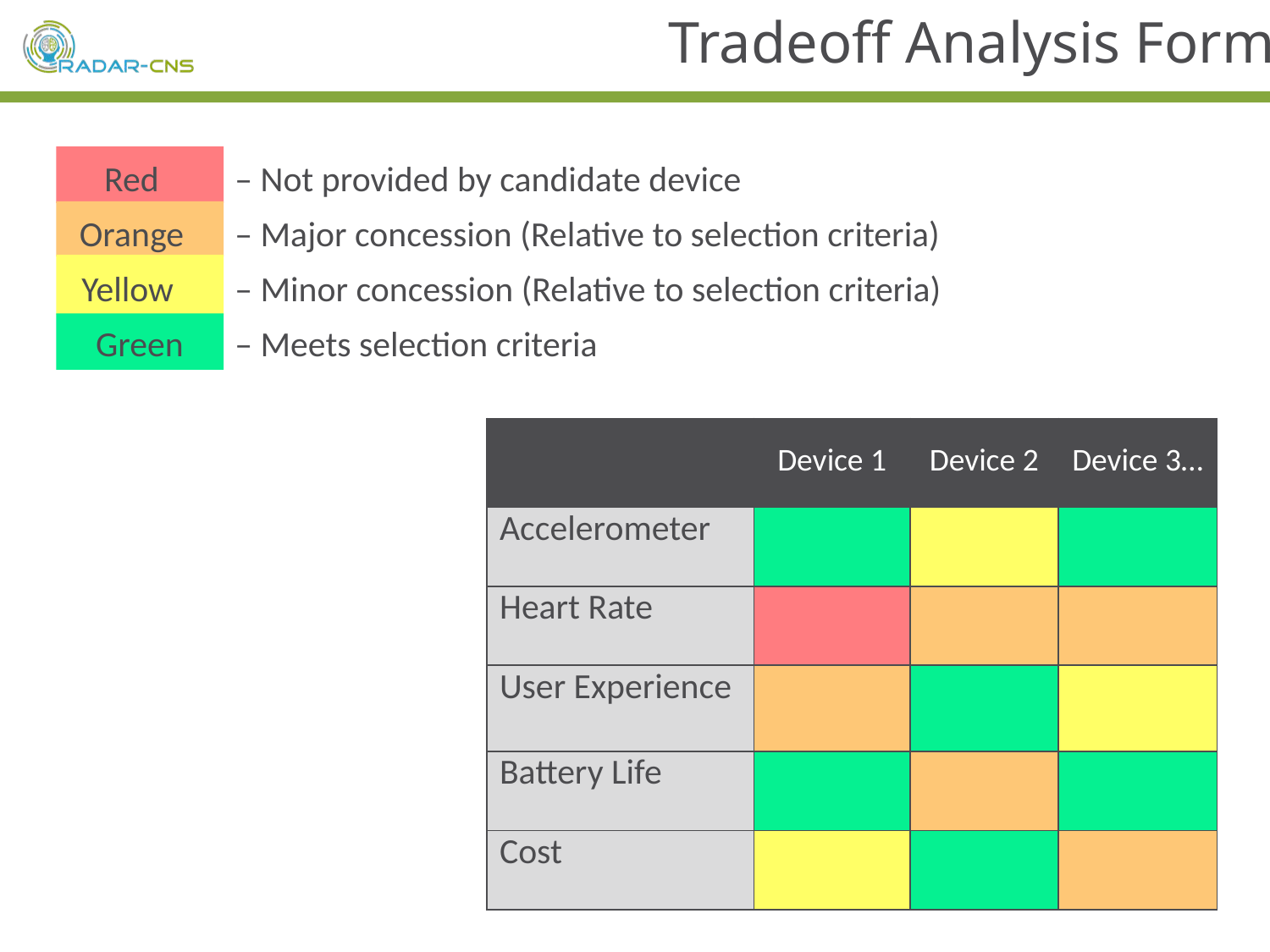

# Tradeoff Analysis Format
– Not provided by candidate device
– Major concession (Relative to selection criteria)
– Minor concession (Relative to selection criteria)
– Meets selection criteria
Red
Orange
Yellow
Green
| | Device 1 | Device 2 | Device 3… |
| --- | --- | --- | --- |
| Accelerometer | | | |
| Heart Rate | | | |
| User Experience | | | |
| Battery Life | | | |
| Cost | | | |

## Slide 4
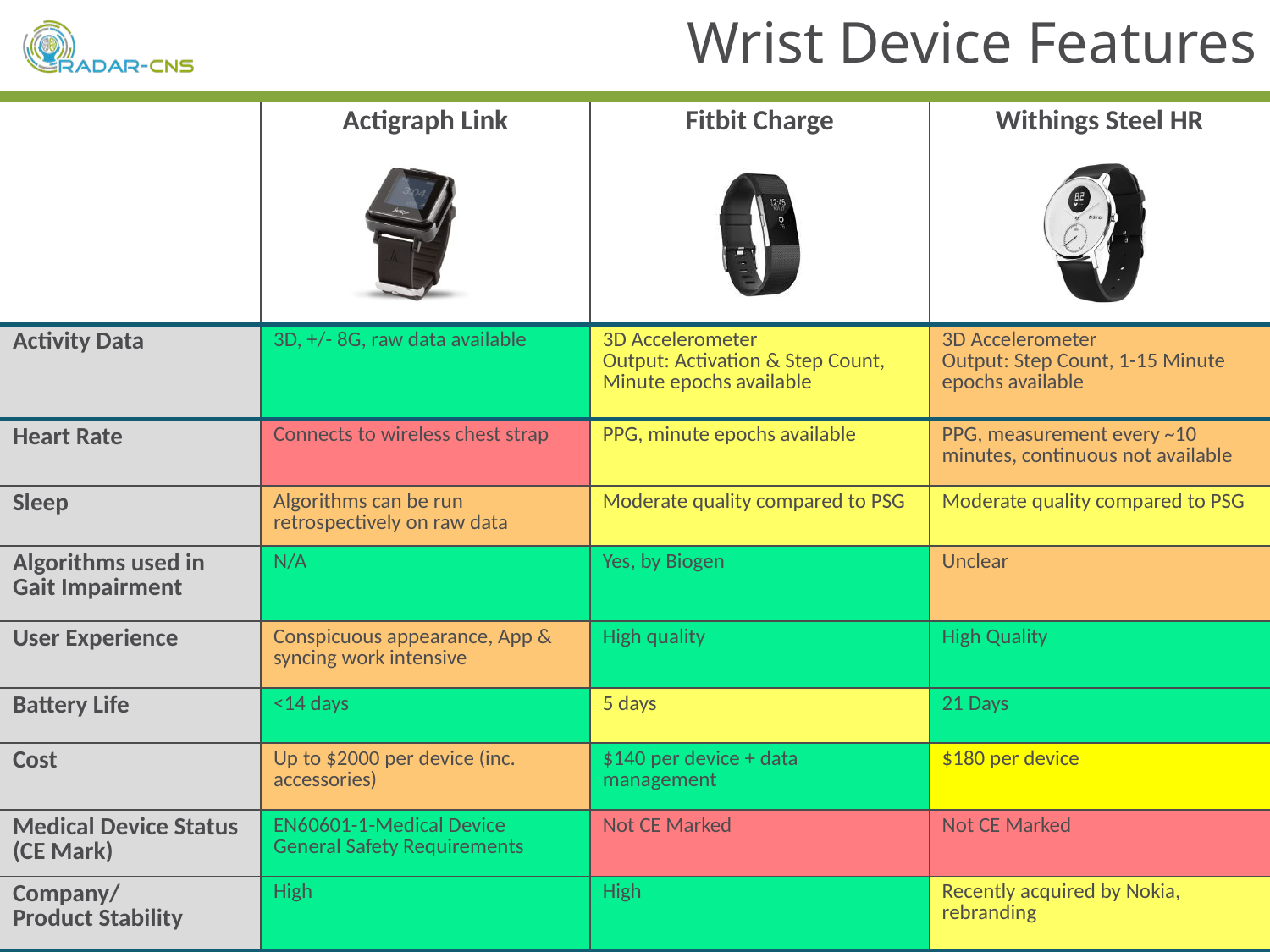

# Wrist Device Features
| | Actigraph Link | Fitbit Charge | Withings Steel HR |
| --- | --- | --- | --- |
| Activity Data | 3D, +/- 8G, raw data available | 3D Accelerometer Output: Activation & Step Count, Minute epochs available | 3D Accelerometer Output: Step Count, 1-15 Minute epochs available |
| Heart Rate | Connects to wireless chest strap | PPG, minute epochs available | PPG, measurement every ~10 minutes, continuous not available |
| Sleep | Algorithms can be run retrospectively on raw data | Moderate quality compared to PSG | Moderate quality compared to PSG |
| Algorithms used in Gait Impairment | N/A | Yes, by Biogen | Unclear |
| User Experience | Conspicuous appearance, App & syncing work intensive | High quality | High Quality |
| Battery Life | <14 days | 5 days | 21 Days |
| Cost | Up to $2000 per device (inc. accessories) | $140 per device + data management | $180 per device |
| Medical Device Status (CE Mark) | EN60601-1-Medical Device General Safety Requirements | Not CE Marked | Not CE Marked |
| Company/ Product Stability | High | High | Recently acquired by Nokia, rebranding |

## Slide 5
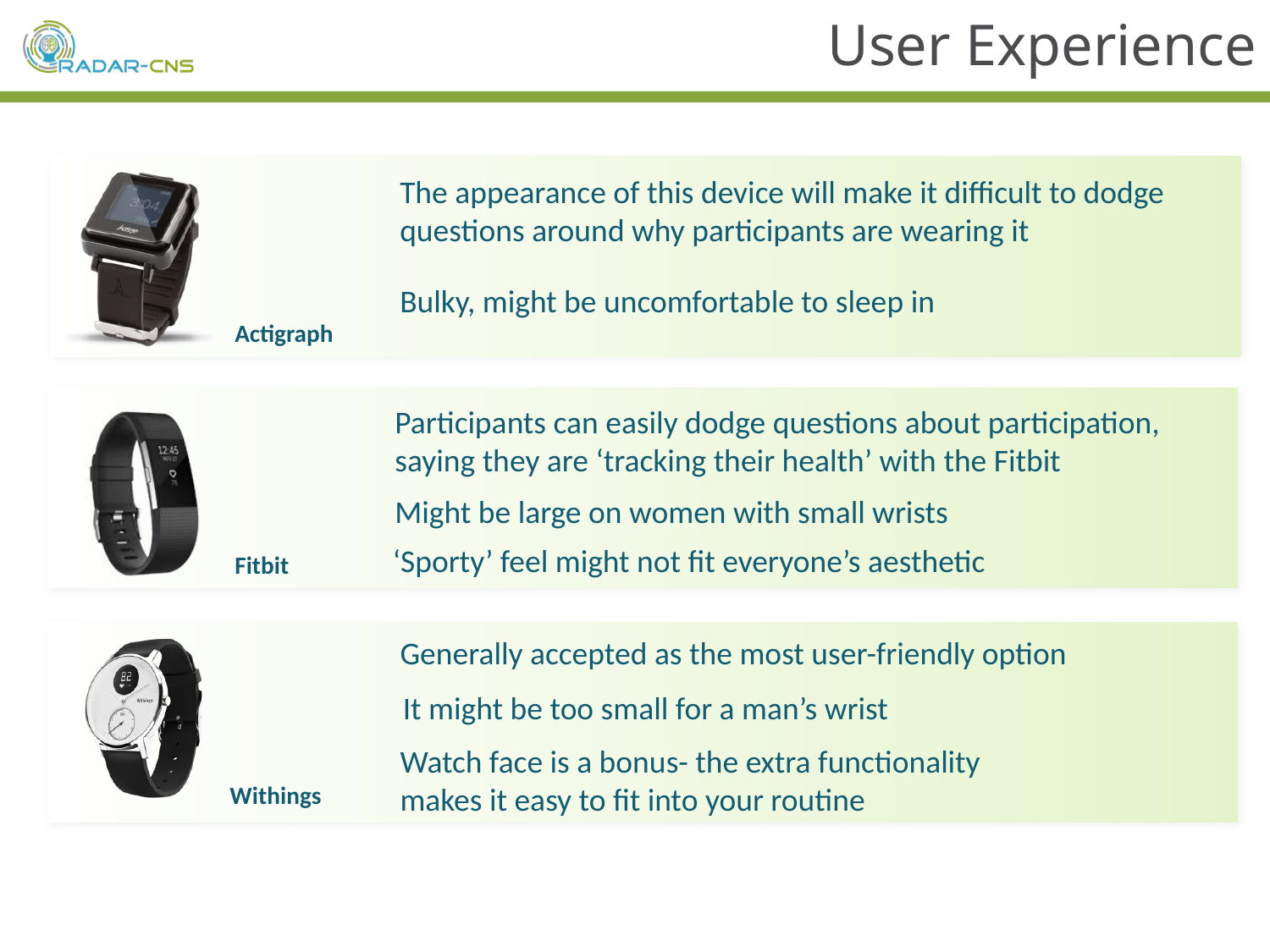

# User Experience
The appearance of this device will make it difficult to dodge questions around why participants are wearing it
Bulky, might be uncomfortable to sleep in
Actigraph
Participants can easily dodge questions about participation, saying they are ‘tracking their health’ with the Fitbit
Might be large on women with small wrists
‘Sporty’ feel might not fit everyone’s aesthetic
Fitbit
Generally accepted as the most user-friendly option
It might be too small for a man’s wrist
Watch face is a bonus- the extra functionality makes it easy to fit into your routine
Withings

## Slide 6
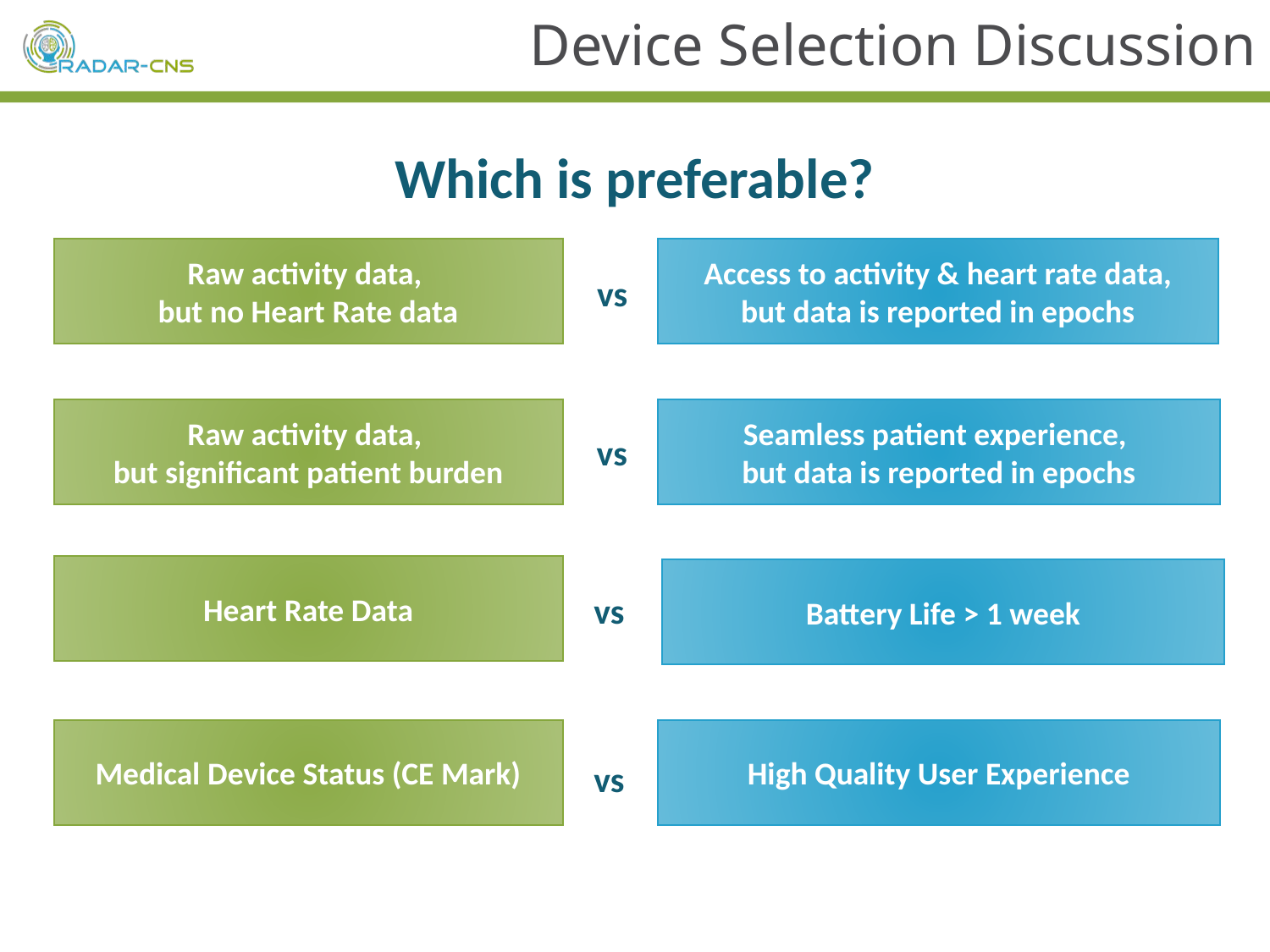

# Device Selection Discussion
Which is preferable?
Raw activity data,
but no Heart Rate data
Access to activity & heart rate data,
but data is reported in epochs
vs
Raw activity data,
but significant patient burden
Seamless patient experience,
but data is reported in epochs
vs
Heart Rate Data
Battery Life > 1 week
vs
Medical Device Status (CE Mark)
High Quality User Experience
vs

## Slide 7
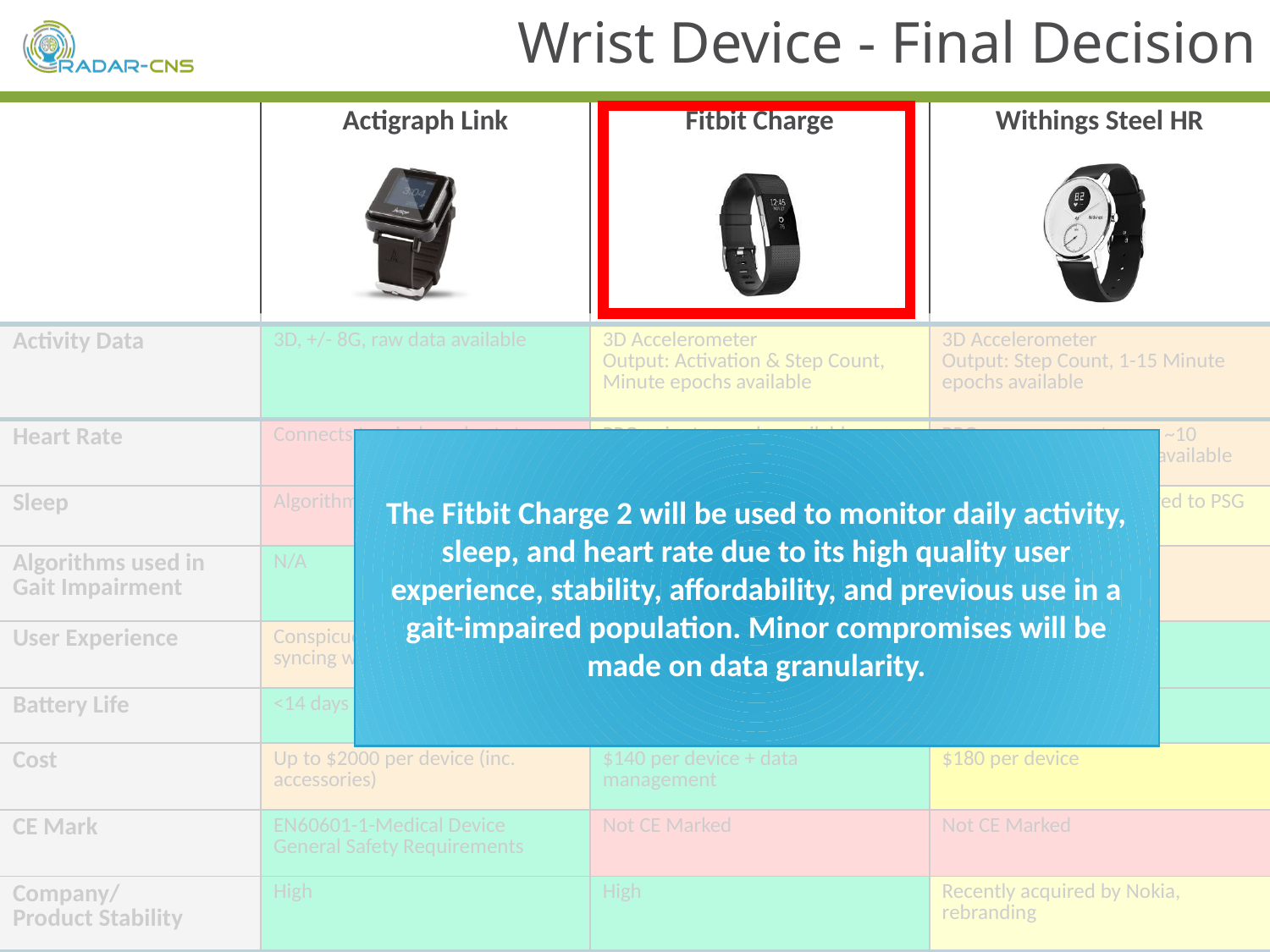

# Wrist Device - Final Decision
| | Actigraph Link | Fitbit Charge | Withings Steel HR |
| --- | --- | --- | --- |
| Activity Data | 3D, +/- 8G, raw data available | 3D Accelerometer Output: Activation & Step Count, Minute epochs available | 3D Accelerometer Output: Step Count, 1-15 Minute epochs available |
| Heart Rate | Connects to wireless chest strap | PPG, minute epochs available | PPG, measurement every ~10 minutes, continuous not available |
| Sleep | Algorithms can be run later | Moderate quality compared to PSG | Moderate quality compared to PSG |
| Algorithms used in Gait Impairment | N/A | Yes, by Biogen | Unclear |
| User Experience | Conspicuous appearance, App & syncing work intensive | High quality | High Quality |
| Battery Life | <14 days | 5 days | 21 Days |
| Cost | Up to $2000 per device (inc. accessories) | $140 per device + data management | $180 per device |
| CE Mark | EN60601-1-Medical Device General Safety Requirements | Not CE Marked | Not CE Marked |
| Company/ Product Stability | High | High | Recently acquired by Nokia, rebranding |
The Fitbit Charge 2 will be used to monitor daily activity, sleep, and heart rate due to its high quality user experience, stability, affordability, and previous use in a gait-impaired population. Minor compromises will be made on data granularity.

## Slide 8
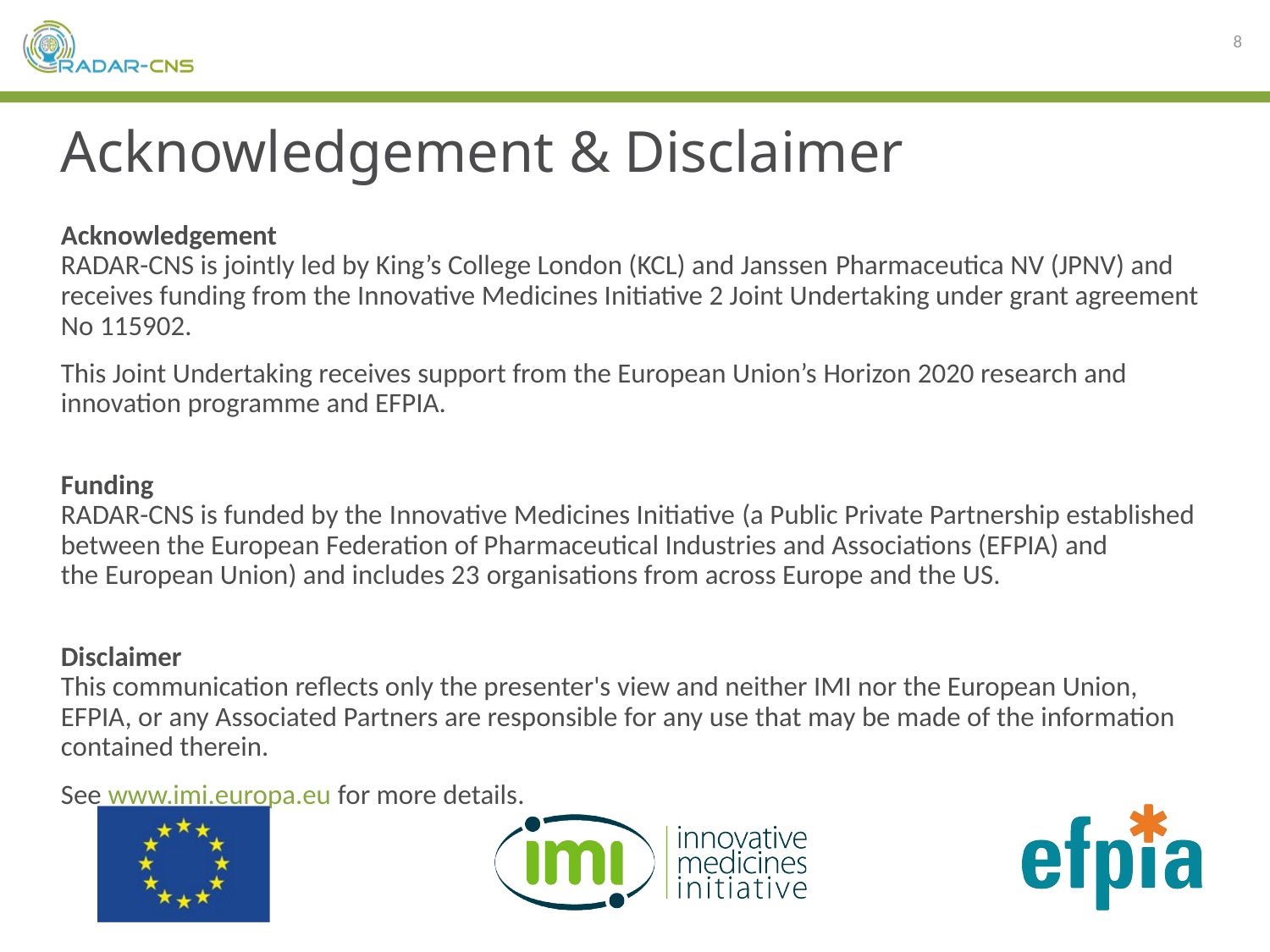

8
# Acknowledgement & Disclaimer
Acknowledgement
RADAR-CNS is jointly led by King’s College London (KCL) and Janssen Pharmaceutica NV (JPNV) and receives funding from the Innovative Medicines Initiative 2 Joint Undertaking under grant agreement No 115902.
This Joint Undertaking receives support from the European Union’s Horizon 2020 research and innovation programme and EFPIA.
Funding
RADAR-CNS is funded by the Innovative Medicines Initiative (a Public Private Partnership established between the European Federation of Pharmaceutical Industries and Associations (EFPIA) and the European Union) and includes 23 organisations from across Europe and the US.
Disclaimer
This communication reflects only the presenter's view and neither IMI nor the European Union, EFPIA, or any Associated Partners are responsible for any use that may be made of the information contained therein.
See www.imi.europa.eu for more details.
